# A Patient with Unilateral Tibial Aplasia and Accessory Scrotum: A Pure Coincidence or Nonfortuitous Association?

**DOI:** 10.1155/2010/898636

**Published:** 2010-02-03

**Authors:** Zoran Gucev, Marco Castori, Velibor Tasic, Nada Popjordanova, Arijeta Hasani

**Affiliations:** ^1^University Pediatric Hospital, Division for Endocrinology and Genetics, Vodnjanska BB, 1000 Skopje, Macedonia; ^2^Medical Genetics, Experimental Medicine Department, “Sapienza” University, S. Camillo-Forlanini Hospital, Rome 0085, Italy

## Abstract

Tibial aplasia is an uncommon lower limb malformation that can occur isolated or be part of a more complex malformation pattern. We describe a 9-year-old boy born after uneventful pregnancy and delivery. Family history was negative for maternal diabetes and other malformations. The patient presented with left tibial aplasia and homolateral prexial foot polydactyly. He also displayed enamel dysplasia and bifid scotum with cryptorchidism. Literature review failed to identify a significant syndromic association between lower limb defects of the tibial type and the genital anomalies reported here. The combination of tibial aplasia with midline genital malformations further supports the hypothesis that the tibial ray development mirrors the morphogenetic process of the radial structures. Accordingly, the malformation pattern observed in the present patient may be pathogenetically explained by an insult occurring during late blastogenesis.

## 1. Introduction

Tibial hemimelia (TH) is a deficiency of tibia and relatively intact fibula. TH may be found as an isolated anomaly or be associated with other skeletal and extraskeletal malformations. TH can be a part of the split-hand/foot malformation with long-bone deficiency (SHFLD) in which the clinical manifestations range from no malformation to ectrodactyly and tibial hypoplasia or aplasia with or without associated anomalies. Other, more complicated malformation complexes or syndromes may also include TH as an important part: the Gollop-Wolfgang complex, triphalangeal thumb-polysyndactyly syndrome, VACTERL or Langer-Giedion syndrome [1]. 

We here describe a 9-year-old boy with TH, polydactyly, and genital anomalies: accessory scrotum and cryptorchidism.

## 2. Clinical History

**Figure 1:**
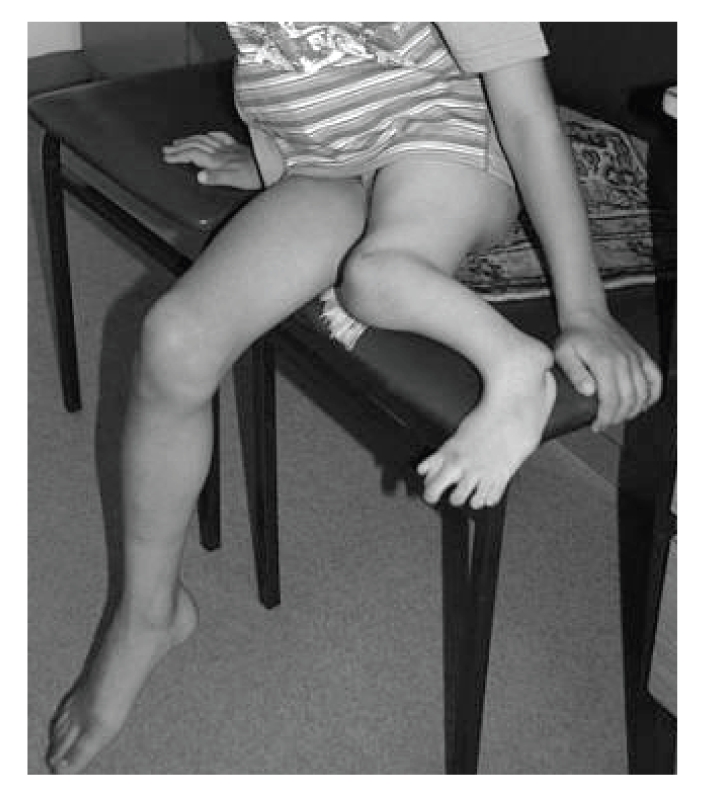
Patient: mesomelic shortening of the left leg with internal rotation of the homolateral foot. The great toe is hypoplastic and bifid. The lateral malleous is protruding as a result from disproportionate development of the tibia and fibula.

**Figure 2:**
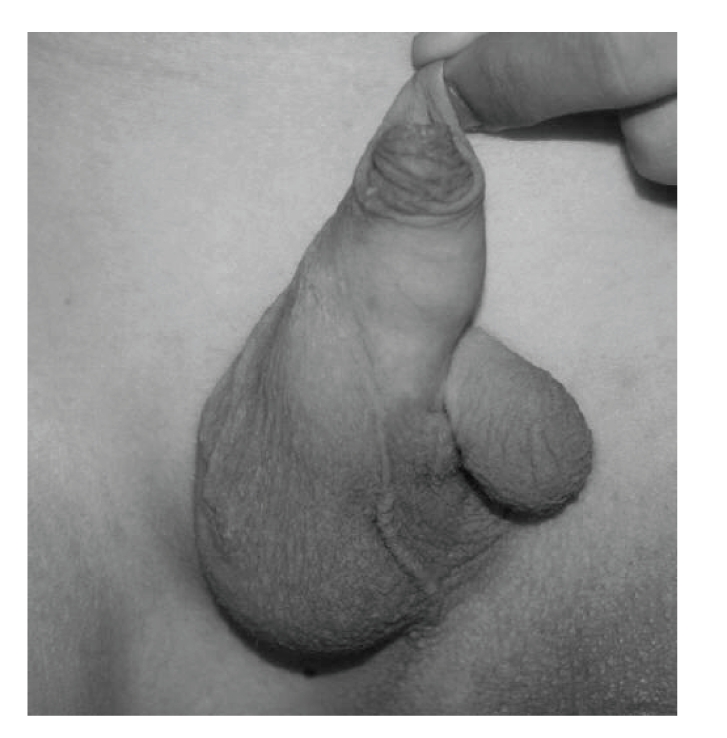
Accessory scrotum originating from the normally developed scrotal sac.

**Figure 3:**
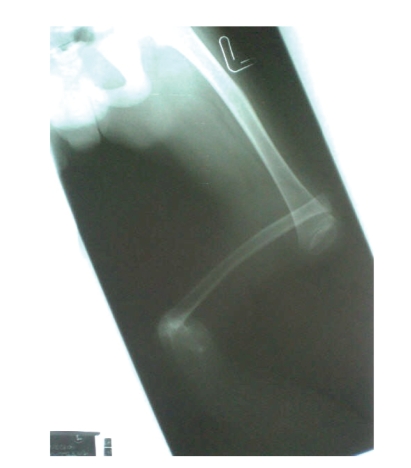
X-ray: complete agenesis of the left tibia, shortened and thickened fibula, and hypoplastic distal femoral epiphysis.

**Figure 4:**
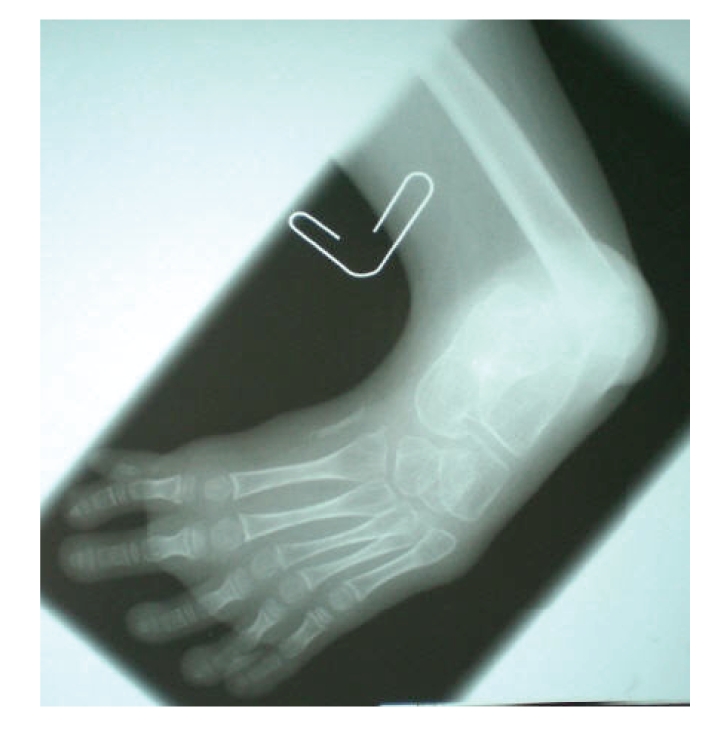
The first metatarsal bone is smaller and longer as resulting from duplication of a more posterior ray. There is a rudimentary accessory metatarsal bone on the tibial side. The great toe is hypoplastic and triphalageal, while a small monophalangeal extradigit is visible on the tibial side of the first toe. The navicular and cuboid bones are unossified.

The proband was a 9-year-old boy, first child of a 20-year-old mother and her healthy and unrelated 28-year-old husband. He was born at term, after uneventful pregnancy and delivery. In particular, pregnancy history was negative for maternal diabetes, vaginal spotting and/or hemorrhages, hyperthermia, radiation exposure, infections, drug and alcohol abuse. Family history was negative for miscarriages and malformations. The proband and his father were heterozygous carriers of beta-thalassemia. Early psychomotor development was normal. In fact, he rolled over at 4 months, stand alone at one leg at 10 months, walked unsupported with prothesis at 15 months, and said first words at 10 months. At time of evaluation, he presented as a well-oriented boy who socialized appropriately for his age. Mentation was normal. On physical examination, mesomelic shortening of the left lower limb with tibial deviation (internal rotation) of the homolateral foot was noted. The great toe was hypoplastic and bifid, while other toes were apparently unremarkable. The lateral malleous was protruding thus suggesting a disproportion between the development of the tibia and fibula (Figure 1). Further examination revealed bifid scrotum and left-sided cryptorchidism (Figure 2). Total X-ray examination revealed complete agenesis of the left tibial with a shortened and thickened fibula and hypoplastic distal femoral epiphysis (Figure 3). This radiographic appearance can be classified as tibial aplasia type 1a, using the Jones classification system (i.e., tibia not seen, hypoplastic lower femoral epiphysis) [2]. The left foot had what follows. First metatarsal bone was smaller and longer than expected and resembled as resulting from duplication of a more posterior ray; there was a rudimentary accessory metatarsal bone on the tibial side; the great toe was hypoplastic and triphalangeal, while a small monophalangeal extradigit was visible on the tibial side of the first toe; the navicular and cuboid bones were unossified (Figure 4). No additional skeletal anomaly was noted. Heart and kidney ultrasound excluded any additional anomaly. Peripheral lymphocyte karyotype and plasma aminoacids level were normal.

## 3. Discussion

The patient presented here recapitulated the spectrum of malformations which belong to the tibial developmental field defect. In particular, we observed agenesis of the tibia, shortening of the homolateral fibula, hypoplasia of the great toe and distal femoral epiphysis, and tibial deviation of the foot. Interestingly, duplication of the first ray with a homeotic change of metatarsal I towards II was also observed. The paradoxical concurrence of deficiency (tibial hypo/aplasia, femoral distal hypoplasia, and first ray deficiency on foot) and duplication (preaxial polydactyly and splitting of the distal femur) is not a rare phenomenon within the spectrum of the tibial developmental field defect. In fact, for example, among the 25 patients with tibial developmental field defect and additional features of the VACTERL association reviewed by Jones et al. [2], this combination is registered in 7 cases [3–6]. In addition, tibial deficiency and great toe duplication may be observed in the maternal diabetes syndrome [7, 8]. This phenomenon is amplified to the extreme in the Laurin-Sandrow syndrome (mirror hand and feet), where agenesis of the tibial ray is substituted by mirror duplication of the fibular structures [9, 10]. 

Tibial hypo/aplasia can be observed in approximately 100 distinct clinical entities [11, 12]. The association of unilateral tibial developmental field defect and accessory scrotum does not fit well with any of the listed conditions. In addition, the sporadicity and striking unilaterality of the observed malformation pattern moves against a germline mutation. In fact, in the clinical practice, unilateral isolated longitudinal and transverse limb defects are usually associated with a very low recurrence risk for the parental couple as well as for the affected individual. In the present patient, the association with bifid scrotum seems to be not sufficient to define a novel malformation syndrome. In fact, a casual association is still possible. However, the combined frequency of two so-rare malformations (i.e., tibial aplasia and bifid scrotum) makes very unlikely this hypothesis. Genital anomalies have been but infrequently described. Genital hypoplasia with absent labia minora and a very small clitoris have been described in a girl [13], as well as vaginal agenesis [14]. In male micropenis [15], bilateral cryptorchidism has been reported. So far there are no reports of associated bifid scrotum and TH. Additional studies investigating the molecular interactions modulating the limb development are needed to clarify this point. 

On a pathogenetic perspective, the association of gross lower limb malformation and midline genital anomalies may shed some light on the timing of the dysmorphogenetic process leading to the observed phenotype. Severe transverse and longitudinal limb defects are considered monotopic defects of blastogenesis. The blastogenesis refers to the events comprised from fertilization to the end of gastrulation in the intrauterine life (i.e., first four weeks). Hindlimb buds appear later than the forelimbs, but they can be usually seen at the 28th day after fertilization [16], therefore, still within the blastogenesis period. Interestingly, a significant association between lower limb malformations (including tibial hypo/aplasia) and extrophy of the cloaca, which is a prototypic monotopic field defect, has been recently outlined [17]. This suggests that, in our patient, the combination of tibial field defect and bifid scrotum may represent the nonrandom association of anomalies resulting from perturbation of adjacent structures (i.e., lower limb and genitalia) by a single dysmorphogenic event acting during late blastogenesis. This hypothesis is partly supported by the existence of additional patients with tibial defects and additional midline genital anomalies.

## References

[1] Castori M, Rinaldi R, Cappellacci S, Grammatico P (2008). Tibial developmental field defect is the most common lower limb malformation pattern in VACTERL association. *American Journal of Medical Genetics A*.

[2] Jones D, Barnes J, Lloyd-Roberts GC (1978). Congenital aplasia and dysplasia of the tibia with intact fibula: classification and management. *Journal of Bone and Joint Surgery. British*.

[3] Slavotinek A, Clayton-Smith J, Kerr B (1999). Unilateral tibial aplasia, pre-axial polysyndactyly, vertebral anomalies and imperforate anus. *Clinical Dysmorphology*.

[4] Tüysüz B, Beker BD, Centel T, Üngür S, Lter O (2001). Unilateral tibial agenesia with preaxial polysyndactyly and renal disorder in two patients: a new syndrome?. *Clinical Dysmorphology*.

[5] Evans JA, Greenberg CR (2002). Tibial agenesis with radial ray and cardiovascular defects. *Clinical Dysmorphology*.

[6] Erickson RP (2005). Agenesis of tibia with bifid femur, congenital heart disease, and cleft lip with cleft palate or tracheoesophageal fistula: possible variants of Gollop-Wolfgang complex. *American Journal of Medical Genetics A*.

[7] Martínez-Frías ML, Cucalón F, Urioste M (1992). New case of limb body-wall complex associated with sirenomelia sequence. *American Journal of Medical Genetics*.

[8] Martínez-Frías ML, Urioste M (1994). Segmentation anomalies of the vertebras and ribs: a developmental field defect: epidemiologic evidence. *American Journal of Medical Genetics*.

[9] Mariño-Enríquez A, Lapunzina P, Omeíaca F, Morales C, Rodríguez JI (2008). Laurin-Sandrow syndrome: review and redefinition. *American Journal of Medical Genetics A*.

[10] Kantaputra PN (2001). Laurin-Sandrow syndrome with additional associated manifestations. *American Journal of Medical Genetics*.

[11] Kantaputra PN, Chalidapong P (2000). Are triphalangeal thumb-polysyndactyly syndrome (TPTPS) and tibial hemimelia-polysyndactyly-triphalangeal thumb syndrome (THPTTS) identical? A father with TPTPS and his daughter with THPTTS in a Thai family. *American Journal of Medical Genetics*.

[12] Kaissi AA, Ghachem MB, Necib MN, Chehida FB, Karoui H, Baraitser M (2002). Hypohidrotic ectodermal dysplasia with tibial aplasia. *Clinical Dysmorphology*.

[13] Wechsler SB, Lehoczky JA, Hall JG, Innis JW (2004). Tibial aplasia, lower extremity mirror image polydactyly, brachyphalangy, craniofacial dysmorphism and genital hypoplasia: further delineation and mutational analysis. *Clinical Dysmorphology*.

[14] Steinkampf MP, Dharia SP, Dickerson RD (2003). Monozygotic twins discordant for vaginal agenesis and bilateral tibial longitudinal deficiency. *Fertility and Sterility*.

[15] Baraitser M, Stewart F, Winter RM, Hall CM, Herman S, Nevin NC (1997). A syndrome of brachyphalangy, polydactyly and absent tibiae. *Clinical Dysmorphology*.

[16] Sadler TW (2009). *Langman’s Medical Embriology*.

[17] Jain M, Weaver DD (2004). Severe lower limb defects in exstrophy of the cloaca. *American Journal of Medical Genetics A*.

